# Advances in repair of non-discoid meniscus injuries in children: a narrative review

**DOI:** 10.3389/fped.2025.1674832

**Published:** 2026-01-29

**Authors:** Ye Tian, Yong-Le Shen, Ke-Lin Wang, Da-Wei Guo, Ting-Ting Hou, Tao Ma

**Affiliations:** Shenyang Orthopedic Hospital, Shenyang, China

**Keywords:** children, non-discoid meniscus injury, physical exercise, repair, research progress, vascular distribution

## Abstract

Non-Discoid meniscus injuries in children and adolescents are generally less common than in adults. However, with the increasing number of children participating in physical exercise and the intensification of exercise intensity, the frequency of meniscus injuries is gradually rising. Currently, research on the diagnosis and treatment of meniscus tears in adults is quite advanced, and some progress has also been made in the research on the repair of meniscus injuries in children. Nevertheless, there is a lack of consensus regarding the repair of meniscus injuries in this population. This study reviews relevant literature on the treatment of meniscus injuries in children in recent years, summarizing aspects such as meniscus vascular distribution, injury classification, mechanisms of injury, and repair methods, aiming to provide a reference for the repair of meniscus injuries in children. This narrative review focuses specifically on non-discoid meniscus injuries in children and adolescents; discoid meniscus lesions, which differ from non-discoid lesions in pathogenesis, morphology, therapeutic approach and outcomes, are excluded from detailed discussion.

## 1 Introduction

The meniscus plays a crucial role in maintaining knee joint stability, load sharing, joint lubrication, shock absorption, and coordinating knee joint activities (1, 2). Meniscus injuries are significantly detrimental to the knee joint and can lead to premature post-traumatic osteoarthritis (PTOA) (3, 4). Typically, meniscus injuries in children are less common than in adults (5). However, with the increasing number of children engaging in physical exercise and the rising intensity of these activities, the incidence of meniscus injuries in children is gradually increasing due to a greater appreciation of the significance of meniscal injuries with enhanced clinical suspicion and radiological investigations (6, 7). It is estimated that 6%–8% of young people in the United States suffer from meniscus injuries each year and also a significant rise in the incidence of meniscal lesions in pediatric patient above at the age of 13 in Italy (8, 9). Furthermore, 80%–90% of these injuries in the United States are related to children's participation in sports, with approximately 50% of patients potentially developing knee osteoarthritis (OA) within 10–20 years after the injury (10, 11). Although certain progress has been made in research on the repair of meniscus injuries in children, there remains a lack of consensus regarding the best approaches for such repairs (12, 13). This article summarizes the literature on meniscus vascular distribution, injury classification, injury mechanisms, and repair strategies in children, aiming to provide valuable insights for the management of meniscus injuries in this population. This narrative review focuses on non-discoid meniscus injuries in children and adolescents and excludes discoid meniscus lesions from detailed discussion, because discoid menisci represent a distinct clinical entity with different pathogenesis, morphology, therapeutic approaches and outcomes. For comprehensive discussions of discoid meniscus, readers are referred to dedicated studies (1).

## 2 Vascular distribution

The vascular distribution of the meniscus is particularly important as it plays a crucial role in the healing of meniscus injuries (14, 15). However, the characteristics of meniscus vascularization in children differ from those in adults. From birth, the meniscus begins to vascularize, with the vascularization range of the neonatal meniscus accounting for about two-thirds of the total blood vessels. As age increases (usually starting at 10 years old), meniscus vascularization gradually decreases (13). After 12 years old, the degree of meniscus vascularization approaches that of adults, with the proportion of vascularization around it being 10%–30% (16). It is evident that the vascular distribution of the meniscus in children encompasses a wider range than that in adults.

The blood supply of the meniscus primarily originates from the capillary plexus surrounding the joint capsule and thesynovial tissue (14). Less than one-third of the meniscus is directly nourished by blood vessels, while most of the remaining tissues depend solely on the surrounding diffuse blood vessels for nutrition (17). KIM W et al. demonstrated that the adjacent synovial tissue possesses a rich blood supply, significantly influencing the healing potential of the meniscus (18). Similar to adults, the meniscus in children can be categorized into three vascular regions (19): the peripheral tissue, abundant in blood vessels, is referred to as the “red-red” zone; the central tissue, which receives a diffuse supply, is termed the “white-white” zone; and the area between the two zones is known as the “red-white” zone. Compared to the meniscus in the “red-red” zone, the tissue in the “white-white” zone, characterized by poor blood supply, exhibits comparatively weaker healing capabilities (14, 20). The meniscus in children demonstrates a broad vascular distribution and a rich blood supply. Theoretically, its healing potential following injury is greater than that in adults, providing crucial guidance for treatment method selection.

## 3 Classification

Meniscal tear classification is commonly organized along two complementary axes: an anatomic (zonal) classification that denotes the location of the lesion within the meniscus, and a morphologic classification that describes the tear pattern (21–23). Historically, Cooper et al. proposed a 12-region division of each meniscus, which remains of historical interes (24). However, the International Society of Arthroscopy, Knee Surgery and Orthopaedic Sports Medicine (ISAKOS) meniscal zonal classification is now widely recommended as a contemporary, standardized framework for reporting meniscal pathology and guiding clinical decision-making (23). Therefore, in this review we adopt the ISAKOS zonal classification as the primary anatomic scheme and retain a concise description of Cooper's 12-region approach for historical context; morphologic tear descriptors remain complementary and are used to guide treatment choices. Compared to adults, meniscus injuries in children follow the same classification (25). Although pediatric meniscal pathology includes both discoid and non-discoid types, the present review focuses on non-discoid meniscus injuries and does not address discoid meniscus lesions in detail.

**Figure 1:**
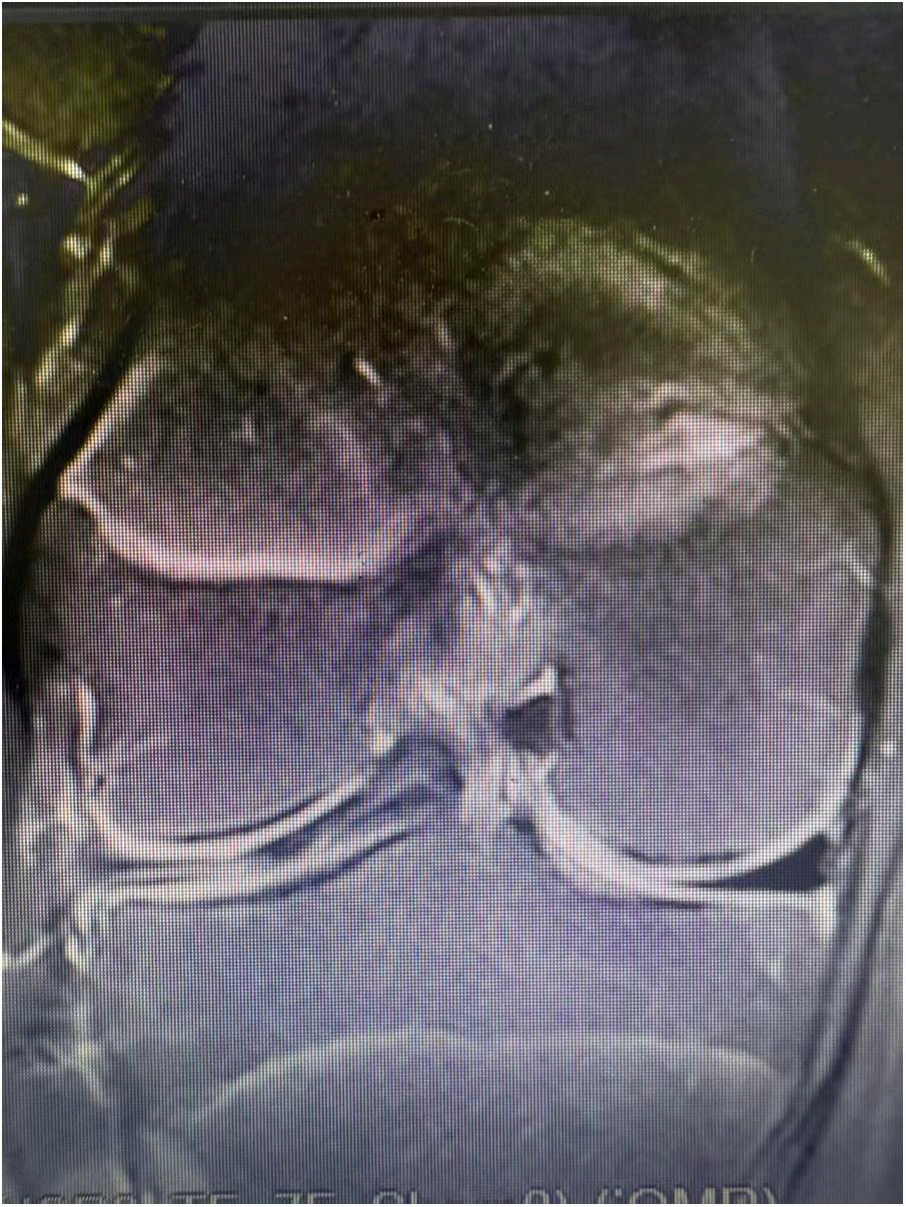
An example of sagittal image showing a lateral meniscus horizontal tear (12 years,female).

**Figure 2:**
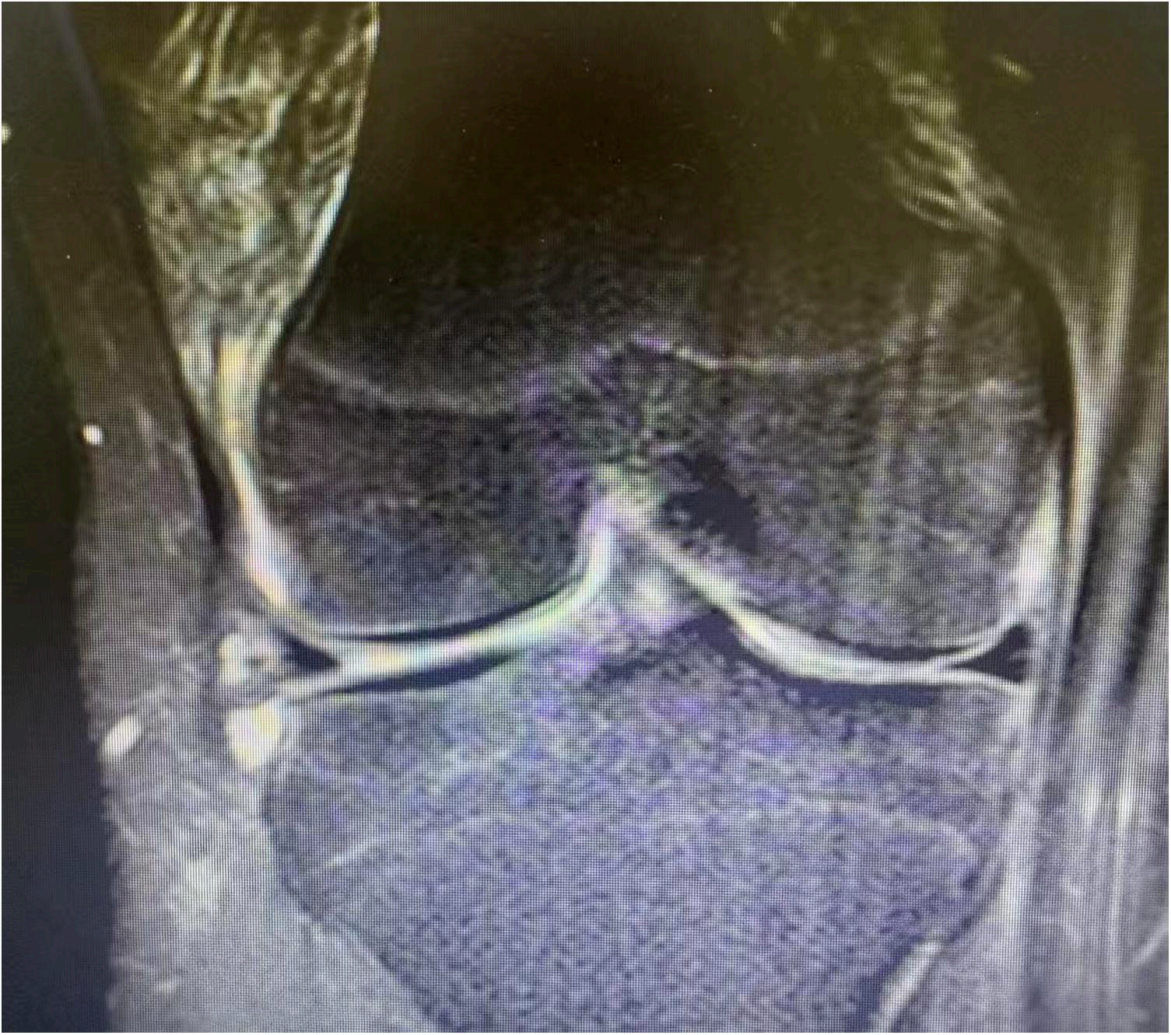
An example of sagittal image showing a lateral meniscus vertical tear (14 years male).

The meniscus is primarily composed of type I collagen fibers arranged in a circular pattern (26). As such, it can be classified based on the direction of the injury in relation to the meniscus plane and the orientation of the collagen fibers, often encompassing horizontal tears (Figure 1), vertical tears (Figure 2), radical tears, parrot-beak, tears, RAMP lesions, hypermobile meniscus and mixed tears.

A long, complete longitudinal tear is typically referred to as a “bucket-handle” tear, while a tear that contains multiple types is classified as a complex tear.

Vertical tears are the most prevalent in children, with approximately 80% occurring in stable knee joints (27). This type of tear aligns with the direction of the collagen fibers, resulting in generally favorable postoperative healing (28). Vertical lesions in unstable knee can progress forward in the meniscus, forming a bucket-handle tear (29).

Radial tears: Radial tears are defined as cleavages that extend approximately perpendicular to the circumferential collagen fibers of the meniscus, interrupting hoop stresses and potentially resulting in meniscal extrusion and altered tibiofemoral contact mechanics. Small, non-displaced radial tears may be managed non-operatively (30). When a radial tear produces mechanical symptoms, functional instability, or extrusion, particularly when located in the midbody or anterior horn, surgical repair is indicated. Horizontal suture configurations are commonly employed for midbody or anterior radial tears to limit extrusion and restore stability. Given the relatively rich peripheral vascularity in children, repair indications may be broader than in adults.

Parrot-beak tears: Parrot-beak tears (also described as flap or oblique marginal tears) involve a short oblique lesion of the meniscal margin that commonly produces a hooked or flipped fragment resembling a “parrot's beak”. Clinically, these lesions may present with focal joint line pain, catching, or intermittent locking. Magnetic resonance imaging can suggest the presence of a parrot-beak lesion, but arthroscopic inspection is often required to determine reparability and to guide intraoperative management. In skeletally immature patients, preservation of functional meniscal tissue is paramount; when repair is technically feasible and tissue quality allows, suture repair should be attempted. If the fragment is irreparable or causes persistent mechanical symptoms, limited partial meniscectomy to remove the symptomatic flap while preserving as much native meniscus as possible is recommended.

RAMP lesions: RAMP lesions refer to injuries at the posteromedial meniscocapsular junction of the medial meniscus, including detachment of the posterior horn from the capsule or meniscotibial structures. These lesions are often associated with ACL injuries and may contribute to persistent posteromedial instability if unrecognized. Given the high rate of concomitant ACL and meniscal injuries in the pediatric population, surgeons should maintain a high index of suspicion for RAMP lesions in patients with ACL tears and perform careful arthroscopic evaluation of the posteromedial compartment. Confirmed RAMP lesions should be repaired, frequently at the time of ACL reconstruction, to restore posteromedial stability and to facilitate meniscal healing.

Hypermobile meniscus: A hypermobile meniscus is a functional condition rather than a discrete tear; it is characterized by abnormal mobility of a meniscal segment due to laxity or detachment of peripheral attachments or roots. Patients with a hypermobile meniscus may present with pain, catching, or mechanical blocking comparable to meniscal tears. Diagnosis often requires arthroscopic evaluation. Management focuses on restoring normal meniscal fixation, arthroscopic repair or fixation of the unstable segment is appropriate when hypermobility correlates with the patient's symptoms. As with other meniscal pathologies in children, strategies should prioritize preservation of viable meniscal tissue.

## 4 Injury mechanisms

Meniscus injuries can either be acute or degenerative. Acute meniscus injuries in adults are typically associated with strenuous exercise and often occur alongside anterior cruciate ligament (ACL) tears or absence, while in the elderly, they frequently coincide with degenerative changes (31). In children, meniscus injuries are generally traumatic, with causes including knee joint rotation, hyperextension, or direct violent impact (32). There is a strong correlation between ACL injuries and meniscus injuries, with this type accounting for about 50% of meniscus injuries in children (15, 33). This association may be explained by the role of ligament integrity in maintaining knee stability: loss of ligamentous stability (for example, chronic ACL deficiency) predisposes to secondary meniscal degeneration and tearing, rather than implying that ligaments protect the meniscus at the exact time of an acute ligament rupture.

Non-discoid meniscus injuries primarily occur in sports such as football, basketball, and wrestling, which involve shear and rotational forces. These injuries are often caused by sudden knee joint bending following foot contact with the ground (34). Discoid meniscus represents a congenital morphological and histological variant with different biomechanical and clinical characteristics compared with non-discoid menisci (35). However, there are currently no reports detailing the specific mechanism behind discoid meniscus tear injuries (36). Detailed discussion of discoid meniscus pathogenesis, clinical features, and management is beyond the scope of this review.

For clarity and to better guide clinical decision-making, pediatric meniscal pathology can be classified into four principal categories: (1) isolated atraumatic lesions; (2) isolated traumatic lesions; (3) combined traumatic lesions (with relevant subtypes); and (4) secondary meniscal injuries developing in the setting of ligamentous insufficiency

### 4.1 Isolated atraumatic lesions

Isolated atraumatic meniscal abnormalities are less common in children than in adults. Imaging appearances that suggest horizontal or degenerative-appearing meniscal signal changes in the pediatric knee should be interpreted with caution because developmental and benign signal variants may mimic true tears on MRI. Pediatric-specific data on the prevalence and optimal management strategies for these atraumatic entities are limited.

### 4.2 Isolated traumatic lesions

Traumatic meniscal tears in children commonly result from rotational forces, hyperextension, or direct impact to the knee (37). Non-discoid meniscal injuries commonly occur during sports with pivoting and cutting movements such as football, basketball and wrestling (37). The tear pattern typically aligns with the meniscal collagen orientation; vertical tears predominate in stable knees and tend to have favorable healing potential after repair (26–28).

### 4.3 Combined traumatic lesions

There is a strong association between ACL rupture and meniscal tears in children, reported to account for approximately 50% of pediatric meniscal injuries (15, 33). This association is best understood in terms of joint stability: the integrity of the knee ligaments is essential for maintaining normal knee biomechanics, and chronic loss of ligamentous support (for example, chronic ACL deficiency) results in instability that increases the risk of secondary meniscal degeneration or tearing. Therefore, clinicians should carefully examine the meniscus in patients with ACL injury or chronic ACL deficiency, since instability-related, secondary meniscal damage may develop over time. Epidemiological studies indicate that the likelihood of combined ACL-meniscal injury may involve the lateral meniscus in the majority of cases (38). RAMP lesions represent a clinically important subtype in the setting of ACL rupture owing to potential instability and implications for repair. The prevalence of ACL-associated ramp lesions in children and adolescents is similar to that in adult populations (15%–24%) (39). Moreover, meniscal tear, displacement, and entrapment are commonly associated with femoral shaft fractures, tibial plateau fractures, and tibial spine avulsion fractures (40).

### 4.4 Secondary meniscal injuries in ACL-deficient knees

Chronic ACL deficiency alters knee biomechanics and increases stress on the meniscus, which can promote secondary meniscal degeneration and complex tear patterns. Biologic responses after meniscal injury (for example, the release of inflammatory mediators such as interleukin-1) and altered load distribution contribute to cartilage loss and progression toward osteoarthritis (41). The present review emphasizes that simultaneous ACL reconstruction may protect the repaired meniscus and is associated with better repair outcomes (42), but detailed pediatric-specific quantitative data on the development, timing, and frequency of secondary meniscal lesions in ACL-deficient knees are not summarized.

## 5 Treatment

The treatment goal for meniscus injuries is to restore the natural biomechanics of the knee joint, relieving discomfort and slowing the progression of osteoarthritis (43). Currently, the approach to treating meniscus injuries in children typically adheres to the principles applied in adults,with options including conservative management and surgical interventions (meniscectomy, suturing, or alternative methods) (44). The optimal clinical treatment plan depends not only on the location, nature, and type of tear but also on the patient's age and potential complications, such as obesity or accompanying cartilage and ligament injuries.

### 5.1 Non-operative management

The mechanical stability of meniscus tears should be assessed primarily on morphological characteristics, such as tear length, width and orientation, involvement of the meniscus, whether the tear is full-thickness, and the presence or degree of displacement or fragment mobility, rather than on vascularity alone. Vascular supply principally determines biological healing potential, and therefore informs the likelihood of repair. However, vascularity does not by itself define biomechanical stability. Accordingly, non-operative management is indicated based on morphology and clinical context.Peripheral meniscus tears measuring less than 10 mm (involving 10% to 30% of the lateral meniscus) are generally considered relatively stable, and non-operative treatment proves to be effective (30). Although the relatively rich vascularization of children's menisci confers greater healing potential for peripheral (“red-red”) tears, the presence of vascularity should be used to estimate biologic healing capacity rather than to infer mechanical stability when selecting conservative vs. surgical treatment. Consequently, non-operative treatment is often employed in the following scenarios: Asymptomatic meniscus tears non-displaced stable menisci (length ≤10 mm, involving 10%–30% of the outer vascular area of the meniscus, with passive displacement <3 mm); Radial tears ≤3 mm.

Conservative treatment typically involves knee joint immobilization, physical therapy, and the use of non-steroidal anti-inflammatory drugs. Prolonged immobilization of the knee can lead to stiffness, muscle atrophy in the affected limb, and ultimately hinder functional recovery (45). While physical therapy and non-steroidal anti-inflammatory drugs may alleviate temporary symptoms, they do not alter the outcomes of meniscus injuries. Furthermore, long-term oral use of non-steroidal drugs poses a risk of peptic ulcers. Therefore, the treating physician should thoroughly inform the child's parents about the risks associated with conservative treatment. If non-surgical methods prove ineffective, surgical intervention should be considered.

### 5.2 Surgical interventions

In pediatric patients, preservation of the native meniscus by arthroscopic repair should be the primary treatment objective and should be attempted in almost all meniscal injuries, unless an intra-operative assessment documents a substantive tissue defect that precludes secure suture fixation (for example, extensive substance loss or irreparably fragmented/degenerative tissue). This repair-first strategy is supported by the greater vascularity and biological healing potential of the pediatric meniscus, and by clinical series reporting high success rates of meniscal repair in children, including a reported arthroscopic repair success rate exceeding 80% in the red-white zone and favorable outcomes across vascular zones (20, 46, 47). Prioritising repair in children contrasts with common adult practice in which meniscectomy is more frequently performed for complex, degenerative, or avascular tears, and aims to minimise the long-term biomechanical and degenerative consequences of meniscal tissue loss (43, 48, 49). Therefore, unless irreparable substance defect is present, meniscal repair should be considered the default surgical strategy for children and adolescents. Meniscus injuries in children possess a greater healing capacity than those in adults (46). Consequently, with the exception of complex types deemed unreparable during arthroscopy, all other types of meniscus tears in children should be considered for surgical repair. Previous studies (17, 50) indicate numerous reports of successful repairs of meniscus injuries in the avascular area among children. The success rate of arthroscopic repair for meniscus tears in the “red-white” zone exceeds 80%, and the indications for repair even extend to the “white-white” zone of one-third of the meniscus. Recently, Cinque et al. demonstrated that regardless of the vascular zone in which the meniscus tear is located, children have a high likelihood of achieving a good repair outcome (20). The duration of non-operative management reported for pediatric meniscal injuries varies in the literature. Several studies recommend an initial structured conservative trial of approximately 12 weeks (51–53). Conversion to operative management is generally considered for patients with acute, large, displaced tears and subacute or chronic tears that cause persistent pain and discomfort, mechanical symptoms, or knee instability, which should be addressed surgically (11).

Large meniscus tears (>40 mm) are often quite unstable, and conservative treatment may not lead to spontaneous repair. Arthroscopic surgical repair has become the primary treatment strategy. The choice of surgical method typically relies on an objective preoperative evaluation. Magnetic resonance imaging (MRI) is the most commonly utilized method for diagnosing meniscus tears and conducting preoperative assessments (40, 54). MRI is the most commonly utilized modality for diagnosing meniscal pathology and for preoperative assessment in children (40, 54). However, pediatric knee MRI can demonstrate a horizontal intrameniscal linear signal that is confined to the meniscal substance and oriented parallel to the meniscal plane. This pattern corresponds to an intrasubstance lesion often described as a Type II lesion (i.e., not extending to the articular surface) and may represent intrameniscal vascular channels or mucoid (intrasubstance) degeneration rather than a surface-extending tear. Such findings, which can be bilateral, should not be interpreted in isolation as an indication for arthroscopic surgery.

Accordingly, imaging must be interpreted in the context of a careful clinical assessment. In the absence of objective clinical symptoms or reproducible mechanical signs, operative intervention is not recommended. When MRI and clinical findings remain equivocal, a stepwise management strategy is advised: initial non-operative treatment; selective repeat imaging only if clinical symptoms persist or worsen; and deliberate exclusion of alternative or concomitant causes. Prior to diagnostic arthroscopy, less invasive confirmatory measures may be considered to help ascertain the pain generator, such as diagnostic intra-articular infiltration with local anesthetic without corticosteroid, thereby reducing unnecessary arthroscopic procedures in children. Diagnostic arthroscopy should be reserved for patients with persistent, clinically significant symptoms, objective mechanical signs, or failure of an adequate course of non-operative management and adjunctive investigations (55).

#### 5.2.1 Partial meniscus resection

Partial meniscectomy should be regarded as a salvage procedure in the pediatric population and avoided whenever repair is feasible. Preservation of native meniscal tissue is a primary objective in children and adolescents because meniscal tissue loss is associated with long-term adverse biomechanical consequences and an increased risk of post-traumatic osteoarthritis. Biomechanical and clinical data cited in this review demonstrate that meniscectomy markedly increases tibiofemoral contact pressures (an increase of 80%–90%) (43), and pediatric series report unfavorable long-term outcomes after meniscectomy (75% symptomatic at 5 years; 80% degenerative imaging changes; 60% dissatisfaction) (49). Total meniscectomy (complete removal of the meniscus) should be avoided and is no longer recommended. Indications for limited (partial) meniscal resection include chronic irreparable tears, complex tears not amenable to repair, tears involving the avascular inner third of the meniscus (“white-white” zone), and symptomatic discoid meniscus lesions that require reshaping (trimming). Consequently, in pediatric patients, meniscectomy should be reserved for true salvage situations in which the meniscal tissue is irreparably damaged, for example, gross tissue fragmentation, severe degeneration with poor tissue quality, or when there is insufficient tissue remaining after multiple failed repair attempts.

Complex meniscal tears are not an absolute contraindication to repair. Advances in arthroscopic techniques, suture devices, and biological augmentation have expanded the reparability of tears in children and adolescents, and successful repairs have been reported even for tears extending into traditionally avascular regions (27, 46). Importantly, meniscal repairs performed concomitantly with ACL reconstruction have been associated with improved healing and superior clinical outcomes compared with isolated meniscal repair, likely due to a more favorable intra-articular biological milieu and mechanical protection provided by ACL reconstruction (42). A meta-analysis comparing meniscal repair with meniscectomy further supports tissue-preserving strategies in younger patients (56).

When meniscectomy is unavoidable, the resection should be limited and tissue-sparing principles followed. Partial meniscectomy is preferred to subtotal or total resection; surgeons should endeavour to preserve the peripheral meniscal rim (approximately 1 cm when technically feasible) to maintain hoop stress transmission and reduce the risk of early degenerative change. Careful intraoperative technique, including staged visualization and judicious use of instruments to avoid inadvertent over-resection, is recommended. Meniscal-preserving alternatives such as meniscal scaffolds or allogeneic meniscal transplantation should be considered in symptomatic meniscal deficiency when the knee is stable (57–60).

Overall, a “repair-first” strategy should guide the management of pediatric meniscal tears, with meniscectomy reserved for irreparable or salvage situations.

#### 5.2.2 Meniscus suture

In the pediatric population a repair-first strategy is recommended. Meniscal suture should be attempted in the vast majority of meniscal tears whenever technically feasible (47). With the exception of tears judged irreparable at arthroscopy, most tear types in children are appropriate candidates for repair because of the comparatively greater vascularity and intrinsic healing potential of the pediatric meniscus (16). Typical indications for meniscal suture in children include acute tears (preferably <8 weeks from injury), tears located in the peripheral vascularized zones (middle and outer third: red-red and red-white), intact bucket-handle tears, and tears occurring in a stable knee (56, 61). Werner et al. found that the proportion of meniscus suture repairs in children surpassed that of meniscectomy, with increases from 2007 to 2011 being 55% and 38%, respectively (61). The earlier the surgical intervention, the higher the cure rate for meniscus tears in children (62). Although the inner avascular “white-white” zone has historically been considered less favorable for repair, in children successful repairs extending into portions of the avascular zone have been reported; therefore, repair may be considered in selected cases after careful intra-operative assessment. In addition to tear chronicity and vascular zone, tear morphology is a key determinant of mechanical stability and suitability for repair. Simple longitudinal/vertical tears, which are reported as the most prevalent pattern in children and generally align with the circumferential collagen fibers, resulting in favorable healing, and small peripheral vertical tears are often relatively stable and are good candidates for repair. By contrast, radial, horizontal, bucket-handle and complex tears may disrupt circumferential fiber continuity, present greater technical challenges, and require specific suture strategies. When repair is not reasonable. Primary suture repair may be not reasonable or technically infeasible in a limited number of circumstances, including: chronic or degenerative tears; complex tears judged irreparable at arthroscopy; severe tissue loss or fragmentation that precludes stable fixation; and certain symptomatic discoid meniscal tears for which resection is indicated. These situations have conventionally been considered indications for partial or subtotal meniscectomy. The choice of suture technique should be guided by tear pattern and location. Vertical sutures are generally preferred for longitudinal tears; horizontal sutures are commonly used for radial tears of the middle and anterior segments to limit extrusion and restore reduction. Needle passage techniques commonly used include inside-out repair for the body, posterior horn and bucket-handle tears; outside-in repair for body and anterior horn tears; and all-inside repair for selected posterior-horn and lateral-body tears. To avoid over-tightening, ensure stability and reduce the risk of secondary ischemia, a suture spacing of approximately 5–7 mm is recommended (63).

When meniscal repair is performed concurrently with anterior cruciate ligament reconstruction, clinical outcomes are generally improved compared with isolated meniscal repair (37). Concomitant ACL reconstruction may enhance meniscal healing and protect the repair from re-injury.

Some surgeons perform a concomitant “healing response” at the time of isolated meniscal repair as a biological augmentation strategy. Techniques described under this approach include trephination (creation of vascular channels) or microfracture of the adjacent subchondral bone to promote ingress of marrow-derived cells and growth factors into the repair site. Although high-quality evidence supporting routine use of these adjunctive procedures is limited, they are commonly applied in clinical practice to enhance meniscal healing, particularly when attempting repair in areas with limited vascularity.

A longer return to sport (RTS) period is seen in the meniscus repair cases (3–4 months postoperatively) when compared with the partial meniscectomy cases (6 weeks postoperatively) (64). If meniscus repair is associated with ACL reconstruction, usually RTS takes longer (8 months) vs. isolated meniscal repair (6 months) (65). However, Lehoczky et al. report similar RTS after ACL reconstruction with or without meniscal repair a prospective cohort study (66).

#### 5.2.3 Alternative approaches

Whether in adults or children, preserving the functional meniscus as much as possible is crucial for the long-term function of the knee joint and serves as the guiding principle for treating meniscus defects. For symptomatic meniscus deficiency with a stable knee joint, post-meniscectomy syndrome, and severe meniscus injuries that cannot be corrected by suture or partial resection, allogeneic meniscus transplantation (MAT) and meniscus scaffold transplantation may be employed. Milachowski et al. (57) first described MAT, which has been proven to relieve knee pain and improve joint function in young patients (58, 60). To maintain or restore the function and mechanical properties of the knee joint, MAT, as a state-of-the-art clinical technology, is increasingly applied to severe or irreparable meniscus injuries.

Ideally, when the meniscus is absent, symptomatic, and the knee joint stable, MAT should be performed since the graft can reduce peak contact pressure between the knee joint surfaces and may slow degenerative changes in articular cartilage. A study demonstrated that in a prospective MAT involving 280 patients under 18 years old (median age 17 years, range 8–18 years), all patients achieved satisfactory treatment outcomes, indicating that MAT can enhance knee joint function and alleviate pain in children and adolescents (59). A Recent systematic review identified three studies including 58 patients (mean age 15.9 years) (67). The lateral meniscus accounted for 82.8% of MAT procedures, and the principal indications were post-meniscectomy syndrome and prior discoid meniscus surgery. All included studies reported improvements in subjective clinical scores and level of sport after MAT. However, the overall complication rate was substantial (reported as 27.5%), and re-operations most commonly consisted of partial meniscectomy, removal of meniscal knots, treatment of chondral defects, and lysis of adhesions (67). Clinically relevant findings from the review include that 41.4% of patients required concomitant procedures (31% related to cartilage damage) and that 15.5% had a previous or concomitant anterior cruciate ligament reconstruction; importantly, outcomes and graft survivorship were reported to be similar between isolated MAT and MAT combined with cartilage procedures (67). However, MAT remains a technically demanding procedure with a relatively high complication and reoperation rate, and the current evidence base is limited. Heterogeneity in graft preservation and construct composition was noted, and the authors emphasized the need for longer-term follow-up and higher-quality studies to determine durability, chondroprotective effects, and optimal patient selection in the pediatric population (67). Most patients treated with MAT return to high-level athletic sports, but participation in high-impact activities should be avoided (68).

Toanen et al. implanted a biodegradable polyurethane scaffold in 155 patients with symptomatic partial meniscus defects (101 medial and 54 lateral) (69). The follow-up results over 5 years indicated that 87.9% of the medial scaffolds and 86.9% of the lateral scaffolds continued to function normally, suggesting that the polyurethane meniscus implant can improve knee joint function in patientswith segmental meniscus defects and relieve pain.

Although allogeneic meniscus transplantation can alleviate pain symptoms, its long-term protective effect on cartilage remains unclear. Additionally, this treatment method has inherent drawbacks, including the risk of disease transmission, immune response, and a limited supply of suitable host tissue. Some scaffolds may also lose their functionality due to contraction, extrusion, and fracture. In summary, these concerns underscore the necessity of long-term follow-up. When assessing the meniscus, the entire knee joint should also be considered to comprehensively evaluate the treatment's effectiveness.

#### 5.2.4 Meniscus regeneration therapy

In recent years, meniscus regeneration therapy has advanced rapidly, with tissue engineering emerging as a potential strategy for promoting meniscus regeneration following meniscectomy. This approach emphasizes the utilization of biological agents and biomimetic natural or synthetic materials, incorporating cells, bioactive factors (both chemical and biological), and injectable or implantable scaffolds to facilitate meniscus healing (70). Among these, growth factors for meniscus tissue engineering, biological materials for meniscus repair, and the reconstruction of the torn sections of the meniscus are critical components of this field.

Popescu et al. (71) injected platelet-rich plasma (PRP) into 30 patients with meniscus tears. The follow-up results after three months indicated that 76.7% of the patients experienced good recovery, significantly enhancing the clinical outcomes for children with meniscus tears. Baboolal et al. (72) mobilized synovial mesenchymal stem cells during surgery using a novel arthroscopic technique. The results demonstrated a substantial increase in the number of functional mesenchymal stem cells, thereby enhancing the repair potential of the joint. Furthermore, significant advancements havebeen made in the repair of avascular meniscus injuries using scaffolds loaded with mesenchymal stem cells and growth plate chondrocytes (73). Zhong et al. employed an injectable extracellular matrix (ECM) hydrogel to repair the meniscus in a rat model of meniscus injury-induced osteoarthritis, achieving favorable results in inhibiting arthritis (74).

Although many studies have demonstrated that implants possess a cartilage-protecting effect *in vivo*, they still face several shortcomings, including poor mechanical properties, significant inflammatory reactions, and rapid degradation of scaffold materials. Therefore, developing a meniscus regeneration scaffold with excellent cell compatibility, high mechanical strength, controllable degradation, and suitable wear resistance remains a considerable challenge. Currently, most of these studies are confined to animal experiments, and substantial progress is needed before achieving real clinical application.

## 6 Conclusion

With the growing popularity of physical exercise, the incidence of meniscus injuries in children is on the rise. There are significant differences in vascular distribution, injury mechanisms, repair methods, and prognoses of the meniscus between children and adults.

Vascular distribution: Children's menisci have arich blood supply. At birth, vascularization nearly covers the entire meniscus; by nine months, it covers 2/3 of the periphery, and the coverage area gradually decreases after the age of ten. In contrast, the blood supply to adult menisci is poor, with only 10%–30% of the outer part of the lateral meniscus receiving blood. The “red-red” zone has the best blood supply, the “red-white” zone is intermediate, and the “white-white” zone has the poorest blood supply.

Injury mechanism: Meniscus injuries in children often occur when the knee joint is suddenly bent during cutting and rotational movements, and they are closely associated with ACL injuries. In adults, these injuries typically result from a torsional force on the loaded knee joint or from contact that induces varus or valgus loading, which is a common complication of ACL injuries.

Repair Method: For children, meniscus suture repair should be prioritized whenever possible, with partial meniscectomy as the secondary option. In adult patients, meniscectomy remains the preferred treatment for complex, degenerative, and avascular meniscus tears, while tears located in the peripheral area of the meniscus should be considered for suture repair. If ACL reconstruction is performed concurrently, the range of the suture can be appropriately expanded.

Cure situation: Children's menisci possess a good blood supply and significant repair potential, resulting in a relatively high cure rate for ACL reconstruction and early surgery. In contrast, the goal of treating meniscus injuries in adults is often to alleviate symptoms such as pain and to slow the degenerative changes of the meniscus and knee joint. The long-term prognosis is poor, and most severe or complex tears are likely to progress to osteoarthritis in later stages.

Therefore, the objectives of treatment are to restore the natural biomechanics of the knee joint, alleviate discomfort symptoms, and slow the progression of osteoarthritis (OA). Among various repair surgeries, meniscus suture provides better treatment outcomes than partial meniscectomy; however, a personalized treatment plan should be tailored to the specific circumstances of each patient. In pediatric patients, the primary consideration should be the protection of the knee joint, allowing those with different meniscus tear locations and sizes to attempt meniscus injury repair. Meniscus repair is typically applicable only to adult meniscus tears with high healing potential, such as peripheral tears. Due to the high degree of vascularization and robust cellular metabolic activity in children's menisci, children possess a greater capacity for repair and recovery.

Importantly, and to emphasize the core clinical principle of this review, surgeons should always prioritize preservation and attempt repair of the meniscus in pediatric patients whenever technically and clinically feasible. With the continuous advancement of surgical techniques, modern instruments, and biological enhancement methods, the success rate of meniscus repair has significantly improved. However, effectively addressing meniscus repair failures or inadequate surgical resections has become a major challenge today. As disciplines such as tissue engineering, biomechanics, genetic engineering, and materials science continue to evolve, Mat and regeneration therapies offer renewed hope for clinically irreparable meniscus injuries.
